# Impact of pregnancy and nutrition on oxidant/antioxidant balance in sheep and goats reared in South Sinai, Egypt

**DOI:** 10.14202/vetworld.2016.801-805

**Published:** 2016-08-03

**Authors:** M. F. Nawito, Amal R. Abd El Hameed, A. S. A. Sosa, Karima Gh. M. Mahmoud

**Affiliations:** Department of Animal Reproduction & Artificial Insemination, National Research Centre, Dokki, Tahrir Street, 12622 Giza, Egypt

**Keywords:** nutrition, oxidant/antioxidant, pregnancy, sheep and goats

## Abstract

**Aim:**

To monitor the effect of nutrition and pregnancy on oxidative status of animals under the arid condition of South Sinai.

**Materials and Methods:**

Blood samples were taken from two groups of animals: The first group retained in farm and fed on concentrate (high diet) and another group grazing natural forage (low diet). Each group was subdivided into pregnant and non-pregnant animals. Blood samples were assayed for their content of malondialdehyde (MDA), total antioxidant capacity (TAC), catalase (CAT), and superoxide dismutase (SOD) enzymes.

**Results:**

MDA level significantly increased in pregnant animals fed either concentrate or grazing low-quality forage and accompanied by a low level of TAC in pregnant grazing animals fed low-quality forage. The activity of CAT decreased in pregnant fed either concentrate or grazing and SOD significant decrease in the pregnant grazing group. These data suggested that the animals might have experienced some degree of oxidative stress and lipid peroxidation and indicating that redox homeostasis was impaired in those pregnant and specially fed on forage rations.

**Conclusion:**

Pregnancy constituted the most oxidative stress facing the grazing and concentrated diet feed sheep and goats under arid and saline conditions of Southern Sinai, Egypt.

## Introduction

Sheep and goats are very important due to their biological factors such as short generation interval, twinning, have short growth periods, and do not require much space. Pregnancy is a physiological process characterized by a drastic increase in energetic and oxygen demands to ensure an adequate fetal development and growth. Thus, both mother and fetus are likely to experience oxidative stress, during pregnancy [1]. In late pregnancy, negative energy balance may be the reason for the development of oxidative stress; increased lipid peroxidation and reduced antioxidant activity are contributing the development of complications in pregnancy [2]. Nutritional levels of animals in pregnancy affected the energy reserves. Furthermore, it declined animal health and production performances. National Research Council (NRC) [3] recommends the ewe’s energy is 1.5 times higher than maintenance during late pregnancy. Fassah *et al*. [4] mentioned that the high metabolic demand in lactation and late pregnancy period also induced oxidative stress.

Oxidative stress is believed to play an important role in maintenance the metabolic activity of some organs and productivity in farm animals [5]. A limited number of conditions were recorded in respect to the effects of oxidative stress in ruminants. It is dangerous because it leaves no clinical signs and the condition is diagnosed by analytical methods to evaluate the defense mechanisms and end products of oxidative stress [6], but “oxidative stress occurs when excess production of pro-oxidants (free radicals) cannot be counteracted by antioxidant mechanisms [7].” Free radicals are highly reactive molecules that include reactive oxygen species and reactive nitrogen species.

Oxidative stress study is a relatively young, and there is a recent development in the evaluation of oxidative stress in farm animals for understanding the fundamental processes involved in metabolic disorders [8]. The activity of oxidative stress biomarkers can be influenced by nutrition. The relationship between diet and oxidative stress has received little research interest during pregnancy in goats [5] and sheep [9]. The oxidative status may be impaired during the pregnancy period. Therefore, the current study aimed to evaluate the effect of diet and pregnancy on oxidative state of goat and sheep during late pregnancy period by detecting the changes in lipid peroxidation and non-enzymatic antioxidant along with enzymatic antioxidant levels in pregnant and non-pregnant animals fed either on concentrate or grazing natural forage in South Sinai.

## Materials and Methods

### Ethical approval

Permission was obtained from Ethics Committee of National Research Center.

### Animals

A total number of 40 sheep (20 fed on grazing and 20 fed on concentrate) and 40 goats (20 fed on grazing and 20 fed on concentrate) from Egyptian local breeds raised in South Sinai were examined for reproductive status by ultrasonographic using 5 MHz linear array probe (SonoVet 600; Medison, Korea). Animals were selected from flocks under grazing conditions, and animals reared on a ration composed of 60% concentrate feed mixture (composed of 30% wheat bran, 15% cottonseed meal, 35% yellow corn, 15% sunflower meal, 3% molasses, 1.5% limestone, and 0.5% salt) plus 40% clover, or clover hay and rice straw according to NRC [10] supplemented twice a day (08:00, 20:00). Accordingly, both groups fed on grazing and fed on concentrated were subdivided into two groups (10 pregnant and 10 non-pregnant). The pregnant sheep and goats were chosen at the last month of pregnancy. The grazing animals were reared at native breeders on low-quality forage which had low protein and high saline content.

### Blood sampling

About 10 ml blood samples collected into heparinized and non-heparinized Vacutainer tubes. Serum collected upon centrifugation at 3000 rpm for 20 min, labeled and stored at −20°C until measuring lipid peroxide malondialdehyde [MDA], total antioxidant capacity [TAC], and catalase (CAT) enzyme activity while heparinized blood for measuring antioxidant enzyme (superoxide dismutase [SOD]) activity.

### Oxidant and antioxidants markers assay

The profiles of lipid peroxidation and oxidative stress markers (TAC and antioxidant CAT enzymes) were detected in serum of sheep and goats. On the other hand, antioxidant enzyme SOD activities were determined in erythrocyte of non-pregnant and late pregnant animals.

Lipid peroxidation product as MDA was assayed using the commercially available kit (Bio-diagnostic, Egypt) according to Ohkawa *et al*. [11] and expressed as nmol/ml. TAC (mM/L), CAT (U/L), and SOD (U/ml) were measured by colorimetric techniques using the Bio-diagnostic kit, Egypt. The method was according to Koracevic *et al*. [12], Aebi [13], and Nishikimi *et al*. [14], respectively.

### Statistical analysis

Data of various parameters were analyzed using computerized software program SPSS version 17.0 using one-way analysis of variance. Comparison of means was carried out by the least square difference. Differences were considered to be significant at p<0.05.

## Result

The lipid peroxidation, non-enzymatic antioxidant levels, and enzymatic antioxidant activities in pregnant and non-pregnant fed on concentrate or grazing low-quality natural forage are presented in Table-1 for goats and Table-2 for sheep.

**Table-1:** Effect of feeding on oxidants/antioxidant parameters in pregnant and non-pregnant goats.

Parameters	Grazing goats		Fed concentrate goats	
	
	Pregnant	Non-pregnant	Pregnant	Non-pregnant
MDA (nmol/ml)	12.80±0.65^a^	11.23±0.98^b^	12.43±0.46^a^	10.44±0.40^b^
TAC (mM/L)	0.53±0.15^b^	0.72±0.07^bc^	0.69±0.14^bc^	1.00±0.09^ac^
CAT (U/L)	215.23±12.93^c^	252.12±12.66^bc^	266.96±16.98^b^	314.10±13.15^a^
SOD (U/ml)	373.80±16.05^c^	419.84±20.71^b^	445.06±18.95^ab^	506.06±8.55^a^

abc Values within the same rows were significantly differ (p<0.05p<0.01). MDA=Malondialdehyde, TAC=Total antioxidant capacity, CAT=Catalase, SOD=Superoxide dismutase

**Table-2:** Effect of feeding on oxidants/antioxidant parameters in pregnant and nonpregnant sheep.

Parameters	Grazing sheep		Fed concentrate sheep	
	
	Pregnant	Non-pregnant	Pregnant	Non-pregnant
MDA (nmol/ml)	16.21±0.99^a^	11.64±0.49^b^	13.28±0.75^b^	11.20±0.41^b^
TAC (mM/L)	0.25±0.11^ac^	0.59±0.07^bc^	0.67±0.21^b^	0.86±0.19^b^
CAT (U/L)	207.93±25.92^bc^	251.98±8.94^ab^	214.67±10.73^bc^	279.63±16.96^a^
SOD (U/ml)	382.48±6.98^a^	449.70±17.22^b^	421.50±10.05^ab^	471.36±13.5^b^

abc Values within the same rows were significantly differ (p<0.05p<0.01). MDA=Malondialdehyde, TAC=Total antioxidant capacity, CAT=Catalase, SOD=Superoxide dismutase

In goats, MDA level significantly increases in both pregnant fed concentrate or grazing than that found in non-pregnant animals fed on concentrate (p<0.01) or grazing (p<0.05). TAC level was lower in pregnant goats fed grazing than the other groups and significantly lower (p<0.01) when comparing with non-pregnant fed concentrate animals. CAT activity significantly decreases in pregnant fed either concentrate or grazing than non-pregnant animals fed on concentrate. Furthermore, CAT activity in grazing pregnant was significant low when comparing with non-pregnant fed concentrate (p<0.001) and pregnant fed concentrate (p<0.05). SOD activity in grazing pregnant was significantly lower value than pregnant fed concentrate (p<0.05) and non-pregnant fed either concentrate (p<0.01) or grazing (p<0.05).

In sheep, MDA level in pregnant sheep either fed on concentrate or grazing showed higher level than non-pregnant, but MDA level in grazing pregnant was significant higher (p<0.01) than that found in pregnant animals fed on concentrate and non-pregnant fed on either concentrate or grazing. TAC level in pregnant sheep fed grazing was the lowest level when comparing with other groups but significantly low with non-pregnant fed concentrate (p<0.01) and pregnant animals fed concentrate (p<0.05). CAT activity showed a significant decrease (p<0.05) in either pregnant fed concentrate or grazing than that found in non-pregnant animals fed on concentrate. SOD value in grazing pregnant was significant low than non-pregnant fed concentrate (p<0.01) and grazing non-pregnant (p<0.05).

## Discussion

The present study recorded that pregnant goats and sheep were exposed to an increased risk of oxidative stress during pregnancy than non-pregnant groups, by the observed increase of MDA value and decrease of TAC level, CAT, and SOD activities, but the oxidative stress was more observed in pregnant grazing than pregnant fed concentrate sheep and goats. Although nutritional factor is one of the oxidative stress, pregnancy constituted the most oxidative stress facing the animals since oxidative stress index (MDA) of both pregnant groups was increased, but TAC level, CAT, and SOD activities were decreased.

Pregnancy is known to be stressful on organisms, which accelerates the production of reactive oxygen species and oxidative stress [15]. The reactive oxygen species are normally neutralized by enzymatic and non-enzymatic defense systems of the organisms. The imbalance between the rate of reactive oxygen species (ROS) production and their neutralization leads to the oxidative stress.

It is well known that high ROS concentrations may lead to oxidative stress and be the cause of many diseases in animals [16]. However, ROS exert a biphasic effect during pregnancy and parturition and, at adequate levels, are fundamental for many physiological events to occur such as embryo implantation [1].

Lipid peroxidation produces a wide variety of aldehydes, which can be formed as secondary products such as MDA [17]. MDA is a specific biomarker of lipid peroxidation. Higher oxidative stress during pregnancy increase blood MDA concentration [18-20]. Several defense mechanisms are available to prevent oxidative damage including scavenging systems as the enzymes glutathione peroxidase and SOD. The SODs convert superoxide radical into hydrogen peroxide and oxygen, whereas the CAT and peroxidases convert hydrogen peroxide into water. In this way, two toxic species, hydrogen peroxide and superoxide radical converted to the harmless product water [21].

Specific biomarkers of lipid peroxidation such as MDA and increased level of MDA are an indication of lipid peroxidation. In our study, MDA level significantly increased in a pregnant animal fed either concentrate or grazing. Similar result obtained in pregnant sheep fed on medium to low-quality forages [6] and pregnant ewes fed traditional or untraditional (salt-tolerant plants) diets [22].

Other studies suggested that MDA concentration increased during late pregnancy [23]. On the other hand, studies in dairy cows during late pregnancy failed to show any significant changes in MDA levels [24]. However, this discrepancy could have been mainly due to individual variations in MDA concentration [24].

The increase in placental progesterone (P4) is accompanied by an augment in blood circulating lipids and MDA, which is a marker of oxidative stress [25]. Mohebbi-Fani *et al*. [6] reported that MDA generation occurred during steroidogenesis. High MDA concentrations found in the placentomes strengthen the hypothesis that pregnancy is characterized by oxidative stress [26], due to the high placental metabolism and steroidogenesis.

TAC level in our study revealed lower value in a pregnant grazing animal, and this result was in agreement with Amer *et al*. [22], who monitored decreased level of TAC in pregnant ewes that fed traditional or untraditional (salt-tolerant plants) diets.

Our results indicated a decrease in CAT activity in pregnant animals fed either concentrate or grazing, and this result was in agreement with Erisir *et al*. [25], who found that erythrocyte activity CAT significantly decreases during pregnancy in Awassi ewes. Furthermore, Öztabak *et al*. [27] reported that plasma CAT activity was lower in pregnant Chios ewes during late pregnancy than in the non-pregnant ewes. Amer *et al*. [22] recorded the same result in CAT activity in pregnant ewes fed traditional or salt-tolerant plants (untraditional) diets.

SOD is well known to be a superoxide radicals’ scavenger which is an essential factor in the protection against free radical damage and is considered the first defense against pro-oxidants [28]. In our study, the lower SOD activity observed in pregnant grazing animals fed low-diet forage was in agreement with Celi *et al*. [5], who recorded decrease of SOD activity in goats fed low diet in energy requirement during pregnancy. Furthermore, Amer *et al*. [22] found that decrease of SOD activity in pregnant ewes fed traditional or untraditional (salt-tolerant plants) diets.

Our results indicated that changes in the nutritional level of the diet had little effect on animals compared with a factor of pregnancy. In this respect, many findings are consistent with the claim that pregnancy is a state of oxidative stress in ruminants [1,6,29-31]. There is also evidence that changes to the nutritional level of the diet had very little or no effect on redox homeostasis in goats during the peripartum period [5]. The well-accepted consensus is that the increase in gestational oxidative stress may be due to the inflammatory process establishing during early pregnancy.

It is essential to consider the oxidative stress indicators are affected by nutrition, and modifications of metabolic substrate may affect their biosynthesis and turnover at the tissue level. It has been shown that feeding dairy cows with high levels of starch increases oxidative stress, possibly due to cellular changes related to oxidative phosphorylation [32]. In sheep, the administration of diets rich in starch had very little effect on redox homeostasis because the reducing power achieved from glycolysis and the pentose phosphate shunt [33]. Sevi *et al*. [34] recorded that grazing adversely affects animal well-being, due to seasonal fluctuations of herbage quality and amount; consequently, grazing animals usually subjected to nutritional stress. Mohebbi-Fani *et al*. [6] refer the oxidative stress accompanied to the low-quality forages due to their deficient in vitamins A and E. Moreover, the ascorbic acid in ruminants may be compromised when gluconeogenesis in the liver is reduced, usually when the diet is primarily roughages. Moreover, Nawito *et al*. [35] reported low levels of trace elements in pregnant sheep and goats reared in South Sinai that is attributed to poor land conditions, low cultivation, and high salinity of this area. So, it is recommended to supply the diet with trace elements to improve antioxidant status which consequently enhances growth performance and animal productivity.

## Conclusions

Pregnancy constituted oxidative stresses on sheep and goats fed concentrate or grazing. The nutritional level of the diet had very little effect on blood oxidant and antioxidant status.

## Authors’ Contributions

KGMM and MFN designed the experimental study, ARA and ASAS performed oxidant and antioxidants markers assay, whereas KGMM and ARA completed data analysis, revision, and writing of the article. All authors read and approved the final manuscript.
